# Supplementary material for: AI-Assistance Body Composition CT at T12 and T4 in Lung Cancer: Diagnosing Sarcopenia, and Its Correlation with Morphofunctional Assessment Techniques
Source: Cancers (Basel). 2025 Oct 8;17(19):3255. doi: 10.3390/cancers17193255 (PMC12524087; doi:10.3390/cancers17193255)
Supplement: Supplementary file 1 [file cancers-17-03255-s001.zip › cancers-3855495-supplementary.pdf]

Suplementary Figure S1: Flow chart diagram of patients selection in our study.

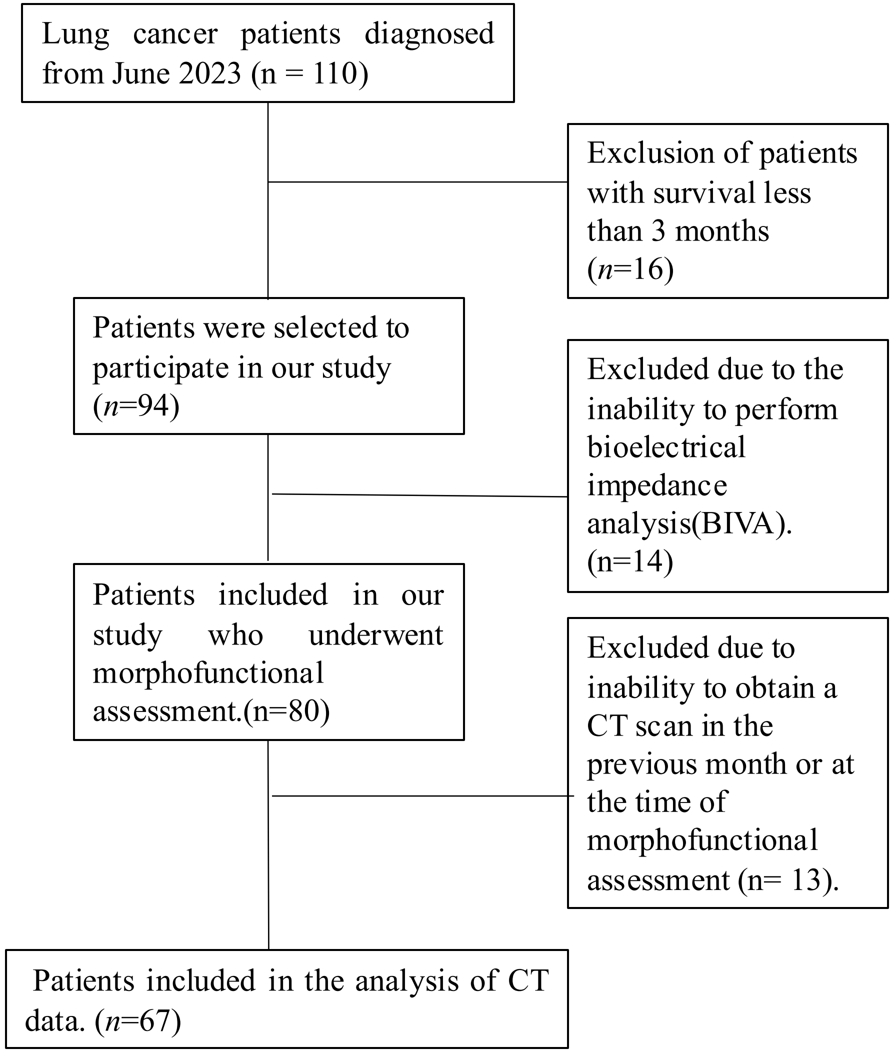

Suplementary Figure S2:

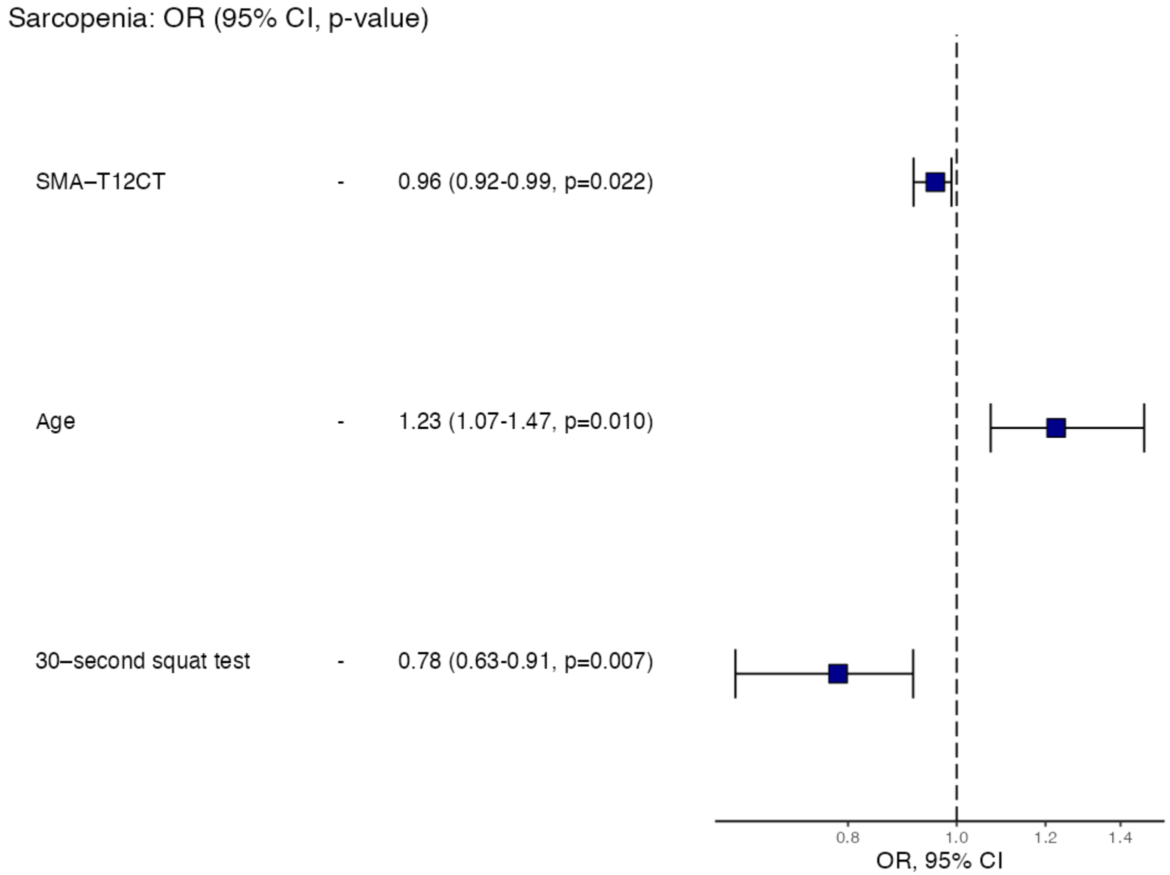

Figure S2. Forest plot showing multivariate odds ratios (ORs) and 95% confidence intervals for SMA–T12CT, age, and 30–second squat test as predictors of sarcopenia. **Abbreviations**: SMI_T12CT: skeletal muscle index at T12 level by CT; OR: odds ratio; CI: confidence interval
